# Spatial variation in the use of reproductive health services over time: a decomposition analysis

**DOI:** 10.1186/s12884-018-1695-3

**Published:** 2018-03-06

**Authors:** Gordon Abekah-Nkrumah

**Affiliations:** Department of Public Administration and Health Services Management, University of Ghana Business School, P. O. Box 78, Legon, Accra Ghana

**Keywords:** Spatial variation, Reproductive health, Decomposition

## Abstract

**Background:**

The paper argues that several Sub-Saharan African countries have recorded marked improvements in the use of reproductive health services. However, the literature has hardly highlighted such progress and the factors responsible for them. The current study uses Ghana as a case to examine progress in the consumption of reproductive health services over the last two decades and the factors responsible for such progress.

**Methods:**

The study uses two rounds (1998 and 2014) of Demographic and Health Survey data from Ghana. Standard frequencies, a logit model and decomposition of the coefficients of the logit model (i.e. Oaxaca-type decomposition) was employed to examine changes in the use of reproductive health services (4+ antenatal visits and skilled attendance at birth) at national and sub-national levels (i.e the four ecological zones of Ghana) between 1998 and 2014 as well as factors explaining observed spatial changes between the two periods.

**Results:**

Descriptive results suggest that the highest level of improvement occurred in resource-poor zones (i.e. northern belt followed by the southern belt) compared to the middle belt and Greater Accra, where access to resources and infrastructure is relatively better. Results from Oaxaca-type decomposition also suggest that women and partner’s education, household wealth and availability and accessibility to health facilities are the key factors explaining spatial variation in reproductive health service consumption over the two periods. Most importantly, the marginal efficiency of investment in women and partner’s education and access to health services were highest in the two resource poor zones.

**Conclusion:**

There is the need to target resource poor settings with existing or new pro-poor reproductive health interventions. Specifically, the northern and southern zones where the key drivers of education and availability of health facilities are the lowest, will be key to further improvements in the consumption of reproductive health services in Ghana.

**Electronic supplementary material:**

The online version of this article (10.1186/s12884-018-1695-3) contains supplementary material, which is available to authorized users.

## Background

Africa’s unfavourable mother and child health outcomes is often the subject of policy and academic discourse. Mortality and morbidity episodes related to women and children constitute a major challenge on the continent. For example, out of 289,000 maternal deaths in 2013, 286,000 occurred in developing countries with 179,000 of these deaths occurring in Sub-Saharan Africa (SSA) alone [1]. In addition, SSA accounted for 49% of global under-five deaths (6,914,300) in 2011, with low birthweight and stunting prevalence being 12% and 40% respectively from 2007 to 2011, [2, 3].

Notwithstanding the above, it is important to emphasise that some SSA counties (e.g. Ghana, Rwnada, Kenya, Tanzania etc) have over the last two decades made tremendous progress in improving mother and child health outcomes through improved access to reproductive and child health services. In Ghana for example, the Maternal Mortality Rate (MMR) reduced by 49% in 2013 [1]. Besides maternal health, child health outcomes have also improved, with infant and under-five mortality declining by 28% and 44% respectively for the period 1998 to 2014 [4]. Also, the percentage of children under-five, who are stunted, wasted or underweight dropped by 16%, 3% and 7% respectively from 2003 to 2014 [4]. Most importantly, these outcome improvements are taking place at a time of increased use of reproductive health services (e.g. antenatal and delivery care), which is considered a key strategy for improving mother and child health outcomes in Ghana [5]. Estimates from the 2014 Ghana Demographic and Health Survey (GDHS) suggest that the percentage of women receiving antenatal care from a skilled provider increased from 82% in 1988 to 97% in 2014, with 4+ antenatal visits (78% in 2008 to 87% in 2014) and health facility deliveries (42% in 1988 to 73% in 2014) improving tremendously [4].

The existing reproductive and child health literature abounds in several studies [6–10] that explain generally, factors that influence the use of reproductive health services such as antenatal and delivery care. Majority of these studies have concluded that individual, household and community level factors influence the use of reproductive health services. What is however not clear in the existing literature is the extent to which these determinants explain changes that have occurred over time. The few studies (i.e. from Ghana, Rwanda and Uganda) [11–13] that have tried to address this issue, have focused their analysis at the national level. This limit one’s ability to understand spatial nuances that may be taking place especially at sub-national levels such the different regions or a group of regions with similar characteristics (ecological zones). In the case of the Ugandan paper [13], the authors mainly computed and compared the probability of using health services conditioned on selected determinants across two periods. Although useful, this approach makes it difficult to know the contributions of the determinants to period changes in the use of such health services.

Thus, the current study uses the Ghana Demographic and Health Survey (GDHS) data to highlight changes in the use of reproductive health services (RHS) (antenatal and delivery care) at the national and ecological zone level between 1998 and 2014 and factors responsible for such changes. Specifically, the study:

1. Examines trends in the use of RHS at the national and ecological zone level between 1998 and 2014.
2. Examine the determinants of use of RHS at the national and ecological zone level using a pooled cross-section of 1998 and 2014.
3. Examine factors contributing to or explaining changes in the use of RHS between 1998 and 2004 at the national and ecological zone level.

The importance of this study is seen in its added value to the existing literature. First, highlighting changes in the use of RHS over the last two decades could constitute a good source of information to guide policy formulation. Secondly, identifying specific factors responsible for any identified changes can be crucial not only for policy formulation, but also appropriate targeting. Finally, the current paper extend the analysis beyond the national level to include the four ecological zones in Ghana. Thus, the study does not only identify factors contributing to changes in the utilisation of RHS over time, but also capture spatial (ecological zone) variation in the contributions of determinants to changes in the use of RHS over time.

## Methods

### Data source

The study used two rounds (1988 and 2014) of GDHS datasets collected by the Ghana Statistical Service and supported by OR/IFC Macro and IFC International Company. The GDHS is nationally representative and based on a two-stage probability sampling strategy. In the first stage, the country was divided into regions and each region into urban and rural areas. Based on the latest available population census sampling frame, primary sampling units (PSU’s) known as clusters were selected from each region in a manner that reflect the rural/urban divide and proportional to the size of the regions. This is done using systematic sampling with probability proportional to size. In the second stage, households are selected from the clusters using systematic sampling with equal probability. Females of reproductive age (15–49) who were either usual members or visitors in the selected households were interviewed. In addition, men aged 15–59 years from a sub-sample of a second or third of total households selected were also interviewed. The survey also collected information on children aged between 0 and 59 months. Information from the survey relevant to this study includes: background characteristics of women and their husbands/partners, reproductive histories, current use of contraceptives, antenatal visits and delivery assistance. The main reason for using the two rounds of the GDHS is to make it possible to examine changes in the use of both antenatal and delivery care as well as examine factors that have contributed to such changes.

### Variable definition and measurement

Given that antenatal and delivery care constitute a major strategy adopted by policy makers in Ghana to improve RHS, we have for the purpose of this study used antenatal and delivery care to capture use of RHS. Antenatal care is captured by a single indicator; whether a pregnant woman had four or more antenatal visits (4+ antenatal visits). Delivery care is also captured by a single indicator; whether a pregnant woman had skilled attendants at birth (doctor, nurse or midwife) when delivering her baby (skilled attendants at birth).

The use of 4+ antenatal visits is based on the WHO recommendation that a pregnant woman needs at least four antenatal visits to be deemed protected from pregnancy-related risk and complications [14, 15]. Thus, we assume that all antenatal visits fewer than four constitute a risk to the pregnant woman. Hence the variable was coded as 1 if a woman had 4+ antenatal visits, else 0 (i.e. a binary choice variable). The strategic value of delivery taking place in a health facility and assisted by skilled birth attendants is that it gives women in labour, access to various delivery services and most importantly, emergency obstetric care (EOC), the absence of which can increase the risk of complicated deliveries. It is therefore assumed that childbirth with the assistance of skilled birth attendants reduces the risk exposure of expecting mothers. Thus, we code any birth assisted by skilled birth attendants as 1 else 0.

Independent variables used in the study are standard determinants of use of reproductive health services also used by several other authors [7–9, 16]. The variables include individual level factors (i.e., woman’s age and age squared, birth order, woman’s level of education and that of her partner, religion and ethnicity), household factors (i.e., household wealth and number of elderly women in the household) and Community factors (i.e., place of residence and availability and accessibility to health services). Bivariate correlation coefficients between the determinants and the outcome variables (results not shown) were calculated to ensure that the determinants where not redundant in the estimated models. Besides, the determinants in the estimated model were introduced into the model in a stepwise fashion to ensure that redundant variables were not included.

Within the RHS literature, availability and accessibility of health services has often been captured using variables such as distance to health facility, category of health personnel and health infrastructure [7, 17, 18]. However, the GDHS data does not have these variables. Thus, we follow existing authors [19–21] by calculating the non-self cluster proportion of households with access to good water (NSCPHGW) and non-self cluster proportion of households with flush toilets (NSCPHGT) as proxies to capture availability and accessibility of health facilities. In principle, the two variables capture and accessibility to and availability of social services such as healthcare within the neighbourhood. For ease of interpretation of the two proxies, determining thresholds (i.e. specific cut-off values) that indicate lower or higher levels of access will be appropriate. However, such cut-off values may be arbitrary and therefore less relevant for purposes of policy. Thus, we follow prior authors [10, 20, 21] by rescaling the two variables to lie between 0 and 1, where values closer to 0 suggest lower levels of access or availability of health services and vice versa. The definition and measurement of all other variables used in the study are contained in Table 1.

**Table 1:** Summary statistics for dependent and independent variables – pooled data for 1998 and 2014

Variables	Obs.	Mean	SD	Variables	Obs.	Mean	SD
4+ Antenatal Visits				Ethnicity			
No	6386	0.214	0.410	Akan (Ref)	6386	0.399	0.490
Yes	6386	0.786	0.410	Ga/Dangme	6386	0.052	0.222
Woman’s Age	6386	30.493	7.229	Ewe and Guans	6386	0.136	0.343
Woman’s Age Sq.	6386	982.076	460.205	North ethnicities	6386	0.393	0.488
Birth order				Others	6386	0.020	0.140
One child (Ref)	6386	0.214	0.410	Number of elderly	6386	1.340	0.665
Two children	6386	0.193	0.395	Household wealth			
Three children	6386	0.161	0.368	Poorest (Ref)	6386	0.315	0.465
Four and above	6386	0.431	0.495	Poorer	6386	0.214	0.410
Woman’s education				Middle	6386	0.183	0.387
No educ (Ref)	6386	0.374	0.484	Richer	6386	0.157	0.364
Primary	6386	0.193	0.395	Richest	6386	0.130	0.336
Secondary	6386	0.404	0.491	Ecological zones			
Tertiary	6386	0.029	0.168	Southern belt (Ref)	6386	0.283	0.451
Partner education				Greater Accra	6386	0.085	0.279
No educ (Ref)	6386	0.296	0.457	Middle belt	6386	0.308	0.462
Primary	6386	0.100	0.300	Northern belt	6386	0.323	0.468
Secondary	6386	0.459	0.498	Rural dummy	6386	0.651	0.477
Tertiary	6386	0.074	0.261	NSCPHGW	6386	0.663	0.340
Missing Partners	6386	0.071	0.256	NSCPHFT	6386	0.100	0.209
Muslim	6386	0.298	0.458	Sample Dummy			
Year Dummy				Skilled Attendance at Birth	6665		
Year 1998	2242						
Year 2014	4144						

Source: Authors’ calculations. Calculations take account of sample weights. Note that the models on skilled attendants at birth is based on slightly different sample per the sample dummy. NSCPHGW and NSCPHFT are the non-self-cluster proportion of households with good water and non-self-cluster proportion of households with flush toilets respectively. Note, partner’s education includes a 5th category (missing partners). This was added to cater for women who do not have partners and would otherwise have been dropped from the regressions. The age variable has also been categorized with number of observations and percentages as follows: 15–19 (2645: 18.6%), 20–24 (2958: 17.3%), 25–29 (2421: 17%), 30–34 (2004: 14.1%), 35–39 (1887: 13.3%), 40–44 (1516: 10.7%) and 45–49 (1308: 9.2%)

**Fig. 1:**
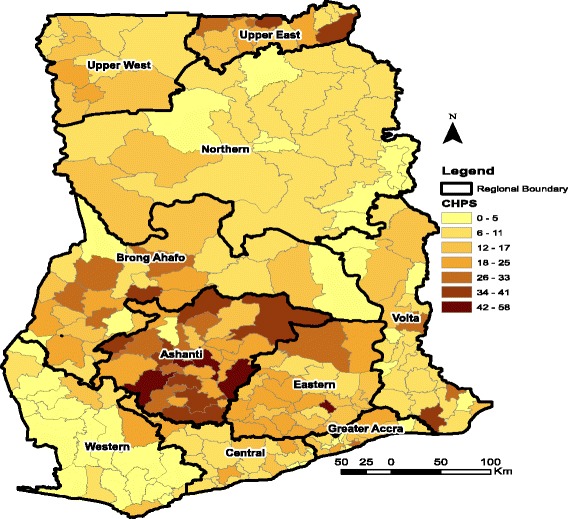
Distribution of CHPS Facilities in Ghana as at 2016. Source: Fig. 1 is constructed by the author using health facility data from GHS. Fig. 1 covers four ecological zones; Northern zone (Upper West, Upper East and Northern Region), Middle zone (Ashanti, Eastern and Brong Ahafo), Southern zone (Western, Central and Volta) and Greater Accra. Note also that CHPS is an acronym for Community Planning and Services; a lower level health facility in Ghana

**Fig. 2:**
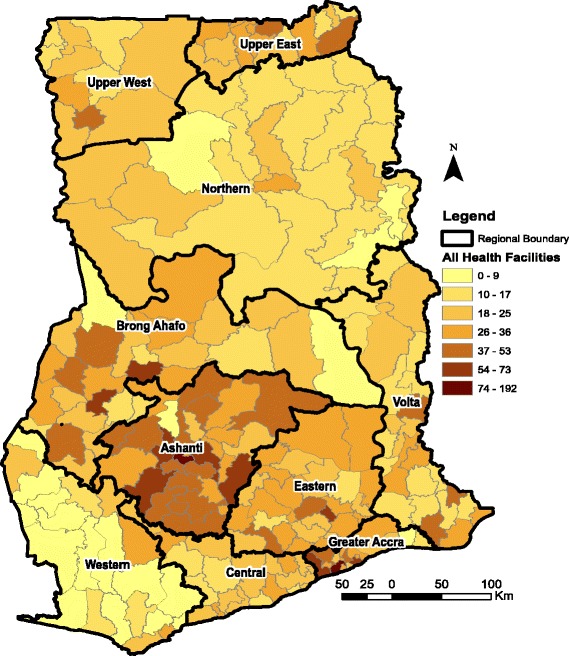
Distribution of Health Facilities in Ghana as at 2016. Source: Fig. 1 is constructed by the author using health facility data from GHS. Fig. 1 covers four ecological zones; Northern belt (Upper West, Upper East and Northern Region), Middle belt (Ashant, Eastern and Brong Ahafo), Southern belt (Western, Central and Volta) and Greater Accra

Four ecological zones (i.e. southern zone, middle zone, northern zone and Greater Accra), based on Ghana’s ten administrative regions are used for the analysis. The southern zone is made up of three regions along Ghana’s coastline; Western, Central and the Volta regions. It is important to emphasise that although Greater Accra region is part of Ghana’s coastline, it has not been added to the southern zone on the basis that it hosts the national capital in addition to having different geographical and climatic conditions from the other regions along the coastline. Hence, Greater Accra is used in the analysis as a separate ecological zone. The middle belt is made up of another three regions located at the middle part of the country; Ashanti, Eastern and Brong-Ahafo regions. The northern belt is equally made up of three regions in the northern part of the country; Northern, Upper East and Upper West regions (see both Figs. 1 and 2 for the ecological zone demarcation).

### Econometric model

#### Estimating determinants of reproductive healthcare use

As indicated in Section 1, a key objective of the paper is examining the determinants of a woman’s decision to use or not to use any of the two reproductive health services. Framing the objective in this manner reduces the utilization decision of a woman into a binary choice set, making it possible to estimate the determinants of use of reproductive health services with a logit model. Thus, if the binary choice set (i.e. having or not having 4+ antenatal visits or skilled birth attendants) is generalized as *J*, and an indirect utility derived from choosing any of the two alternatives as *V*, then the log odds of a woman using any of the two reproductive health services can be expressed as in Eq. 1

1 $$ Ln\left(\frac{P\left({V}_j\right)}{1-P\left({V}_j\right)}\right)={X}_j\beta +{\varepsilon}_j $$

Where, for instance, *V*_*j*_ = 1 if reproductive healthcare is used based on the definition of the variables in Table 1, and *V*_*j*_ = 0 if otherwise. *X *represents a vector of determinants at the individual, household and community level, with *β* being coefficients to be estimated and *ε*, the error term. It is important however to emphasise that the *β*‘s reported in Tables 3 and 4 are marginal effects and not log odds. The use of the marginal effects makes it possible to interpret the *β*‘s as change in the outcome variable as a result of a unit change in the determinants. This notwithstanding, the log odds results have also been reported in Additional file 1: Table S1and Table S2.

#### Decomposition of changes in the use of reproductive health services

To decompose period (1998–2014) changes in 4+ antenatal visits and skilled attendants at birth over their determinants both at the national and ecological zone level, we used the binary analog of the standadard Blinder [22] and Oaxaca [23] decomposition technique developed by Fairlie [24–26]. The use of the Fairlie approach is due to the fact that the standard Blinder-Oaxaca decomposition is not suitable for decomposing changes in a binary variable such as 4+ antenatal visits, and skilled attendants at birth.

Following from Fairlie [24], the decomposition of a nonlinear equation $Y=F\left(X\widehat{\beta}\right),$ can be expressed as:

2 $$ {\overline{Y}}^R-{\overline{Y}}^B=\left[\sum \limits_{i=1}^{N^R}\frac{F\left({X}_i^R{\widehat{\beta}}^R\right)}{N^R}-\sum \limits_{i=1}^{N^B}\frac{F\left({X}_i^B{\widehat{\beta}}^R\right)}{N^B}\right]+\left[\sum \limits_{i=1}^{N^B}\frac{F\left({X}_i^B{\widehat{\beta}}^R\right)}{N^B}-\sum \limits_{i=1}^{N^B}\frac{F\left({X}_i^B{\widehat{\beta}}^R\right)}{N^B}\right] $$

Where ${\overline{Y}}^j$ is the average probability of the outcome of interest for group *j,* N_j_ is the sample size for group *j*, *R* is reference group (i.e. 2014), B is the base group (1998), and $\widehat{\beta}$ a vector of coefficient estimates for group *j,* and *F*, a cummilative distribution function from a logistic distribution. The first term in the bracket represents that part of the gap in the dependent variable that is due to group differences in the distribution of *Xs*. The second bracket represent the gap arising from differences in group processes that determines the level of *Y* as well as the proportion of the group difference captured by unobserved endowments [26]. Thus, Eq. 2 is used to estimate the contribution of period (1988 to 2014) differences in the entire set of determinants to the gap in the dependent variables.

To estimate the independent contributions of individual determinants to the period gap in the dependent variables, coefficients ${\widehat{\beta}}^{\ast }$ from the logit regression for a pooled sample of the two periods are used as in Eq. 3 below, to estimates the contributions of X_1……..._ Xn.

3 $$ \frac{1}{N^B}\sum \limits_{j=1}^{N^B}F\left({\widehat{a}}^{\ast }+{X}_{1j}^R{\widehat{\beta}}_1^{\ast }+{X}_{2j}^R{\widehat{\beta}}_2^{\ast}\right)-F\left({\widehat{a}}^{\ast }+{X}_{1j}^B{\widehat{\beta}}_1^{\ast }+{X}_{2j}^R{\widehat{\beta}}_2^{\ast}\right) $$

As has been the case in many studies, we donot focus on the decomposition of the error term and so not reported in the results.

Additionally, standard errors can be calculated for the decomposition and thereby make it possible to test the significance of the estimated contributions of the covariates. As shown by Fairlie [26], Eq. 3 can be rewritten as Eq. 4 using the delta method to approximate the standard errors.

4 $$ {\widehat{D}}_i=\frac{1}{N^B}\sum \limits_{i=1}^{N^B}F\left({X}_i^{RR}{\widehat{\beta}}^{\ast}\right)-F\left({X}_i^{BR}{\widehat{\beta}}^{\ast}\right) $$

From Eq. 4, the variance of ${\widehat{D}}_i$ can be approximated as Eq. 5 below:

5 $$ Var\left({\widehat{D}}_1\right)=\left(\frac{\delta {\widehat{D}}_1}{\delta {\widehat{\beta}}^{\ast }}\right) Var\left({\widehat{\beta}}^{\ast}\right)=\left(\frac{\delta {\widehat{D}}_1}{\delta {\widehat{\beta}}^{\ast }}\right) $$

Where $\frac{\delta {\widehat{D}}_1}{\delta {\widehat{\beta}}^{\ast }}=\frac{1}{N^B}\sum \limits_{i=1}^{N^B}f\left({X}_i^{RR}{\widehat{\beta}}^{\ast}\right){X}_i^{RR}-f\left({X}_i^{BR}{\widehat{\beta}}^{\ast}\right){X}_i^{BR}$ and *f* is the logistic probability density function.

To estimate Eqs. 3 and 5, one will need a 1 to 1 matching of the observations of the 1998 and 2014 samples. However, it is the case that the 2014 sample is larger than the 1998 sample (see Table 1). To overcome this challenge, we follow Fairlie [26] and draw a large number of random samples (5000) from the 2014 sample and match each of the random samples to the 1998 sample to calculate the decomposition. The reported result is therefore the mean of the repeated calculations.

## Results

### Descriptive results

In this section, we present results on the use of the two reproductive health inputs (4+ antenatal visits and skilled birth attendance) using a pooled sample for two periods (1998 and 2014) at the national level and across the four ecological zones. The results in Table 2 suggest that at the national level, women making 4+ antenatal visits increased by 22.9%, with birth attended by skilled personnel also increasing by 29.8%. The improvement in utilization at the national level is also reflected in utilization at the ecological zone level. The southern zone recorded the highest level of improvement in 4+ antenatal visit (25.2%), followed by the middle zone (23.6%), the northern zone (21.2%) and finally the Greater Accra region (15.5%). In the case of skilled birth attendants, the northern zone recorded the highest increase (36.8%), followed by the southern zone (31.9%), the middle zone (27.4%) and Greater Accra (17.2%).

**Table 2:** Trends in 4+ antenatal visits and skilled birth attendance and selected independent variables

Variables	National		Southern Belt		Capital		Middle Belt		Northern Belt	
	1998	2014	1998	2014	1998	2014	1998	2014	1998	2014
4+ Antenatal	64.86	87.71	62.78	87.95	76.11	91.59	65.77	89.36	59.53	80.7
Skilled Birth Attendants	46.22	75.99	42.12	74.03	75.96	93.18	53.67	81.07	17.2	54.02
Woman’s Education										
No education	29.12	19.07	25.94	15.82	14.76	8.33	21.09	12.86	76.88	56.26
Primary	18.05	17.79	21.29	19.81	15.63	14.21	19.53	18.60	8.66	16.77
Secondary	50.58	56.80	50.74	59.63	63.84	63.78	58.29	63.42	12.95	24.64
Tertiary	2.25	6.34	2.03	4.74	5.78	13.69	1.08	5.13	1.51	2.34
Partner’s Education										
No education	22.77	20.11	14.30	12.65	8.87	6.57	13.86	12.98	77.26	65.76
Primary	7.84	8.85	9.54	10.13	4.56	4.76	8.24	9.19	6.05	10.65
Secondary	60.66	58.87	65.44	66.04	73.67	68.28	71.19	66.91	11.24	16.66
Tertiary	8.73	12.17	10.72	11.18	12.90	20.38	6.71	10.92	5.45	6.93
Household Wealth										
Poorest	20.26	16.09	18.63	7.96	2.33	1.82	16.30	9.59	57.61	68.60
Poorer	17.22	17.41	20.53	24.73	3.78	2.51	19.53	20.90	19.28	14.89
Middle	18.20	20.62	26.52	31.20	4.06	10.30	20.79	22.84	7.32	8.42
Richer	20.12	22.53	20.87	22.94	22.55	29.06	22.40	25.48	8.99	5.13
Richest	24.21	23.35	13.45	13.16	67.28	56.31	20.98	21.20	6.81	2.95
NSCPHGW (Mean)	.5659	.7961	.5066	.7457	.8425	.9384	.5249	.7746	.4822	.7498
NSCPHGT (Mean)	.0874	.2381	.0372	.1425	.3084	.5709	.0564	.2113	.0242	.1083

Source: Calculated by author using data from the 1998 and 2014 Ghana DHS. Note estimates uses sample weights

Table 2 also shows trends in key policy variables such as education, household wealth and availability and accessibility of health services. The northern zone witnessed the greatest level of improvement in women’s education at all levels except for the tertiary level. Women with no education reduced by 20.6%, whiles those with primary and secondary education increased by 8.1% and 11.7% respectively. The southern zone also witnessed appreciable improvements in women’s education with a 10.1% drop in women with no education and an 8.9% increase in women with secondary education. At all levels of women’s education, Greater Accra had the least improvement except that it had the highest increase in women with tertiary education (7.9%). The performance of the northern zone with respect to partner’s education is even more pronounce compared to the other ecological zones. Partners with no education reduced by 11.5%, whiles those with primary and secondary education increased by 4.6% and 5.4% respectively.

In addition, the southern zone showed the strongest improvement in household wealth, with a 10.7% drop in women in the poorest wealth quintile, but a 4.9% and 2.1% increase in the middle and richer quintiles respectively. Whereas the middle zone witnessed some improvement in household wealth, the northern zone exhibited the least progress, recording an increase of 11% in women categorized as the poorest as well as a 3.8% drop in the number of women categorized as richer or richest. On availability and accessibility of health services, the northern zone recorded the biggest increase (0.268) in the mean proportion of women who have access to good water in the neighbourhood compared to the lowest increase (0.096) recorded by the Greater Accra region. On the contrary, Greater Accra recorded the highest increase in the proportion of women in the neighbourhood with access to flush toilets, followed by the middle and southern belts.

### Determinants of use of reproductive health services

In this section, we present the results of the determinants of 4+ antenatal visits and skilled attendants at birth. Although results on log odds are reported in Additional file 1: Table S1 and Table S2, the discussion in this section is based on marginal effects as reported in Table 3 to Table 4. Given that the results are not different from what is known in the existing reproductive health literature, we summerise the results, but highlight key findings, especially at the ecological zone level. The results in Tables 3 and 4 shows that whiles women’s age has a quadratic effect on the two RHS, birth order, being a Muslim woman, number of elderly women in the household and living in a rural area are all significantly negatively correlated with both 4+ antenatal visits and skilled birth attendants. On the contrary, women and partners education, household wealth and access to and availability of health services were significantly positively correlated with 4+ antenatal visits and skilled birth attendants.

**Table 3:** Determinants of 4+ antenatal visits in Ghana – marginal effect estimates

Variables	Ecological Zones				
	National	Southern	Gt Accra	Middle	Northern
Woman’s_Age	0.0223^***^	0.0261^**^	0.0216^**^	0.0246^***^	0.0061
	(0.0054)	(0.0114)	(0.0099)	(0.0089)	(0.0102)
Woman’s age Square	−0.0003^***^	−0.0003^*^	−0.0003^**^	−0.0003^**^	−0.0001
	(0.0001)	(0.0002)	(0.0002)	(0.0001)	(0.0001)
Birth Order: 2nd Birth	−0.0422^**^	−0.0533	−0.0810^*^	−0.0638^*^	0.0210
	(0.0190)	(0.0357)	(0.0435)	(0.0357)	(0.0355)
Birth Order: 3rd Birth	−0.0572^**^	−0.0642	−0.0699	−0.0971^**^	0.0078
	(0.0228)	(0.0453)	(0.0454)	(0.0472)	(0.0384)
Birth Order: 4th Birth	−0.0944^***^	−0.1145^**^	−0.0622	−0.1193^***^	−0.0237
	(0.0214)	(0.0447)	(0.0476)	(0.0396)	(0.0417)
Woman’s Education: Primary	0.0310^***^	0.0388^*^	0.0142	−0.0133	0.0764^***^
	(0.0119)	(0.0212)	(0.0185)	(0.0221)	(0.0254)
Woman’s Education: Secondary	0.0808^***^	0.1096^***^	0.1094^***^	0.0276	0.0702^**^
	(0.0141)	(0.0252)	(0.0322)	(0.0241)	(0.0335)
Woman’s Education: Tetiary	0.1148^***^		0.0595^***^		0.1320^**^
	(0.0326)		(0.0226)		(0.0670)
Partner Education: Primary	0.0336^**^	0.0274	−0.0133	0.0202	0.0698^***^
	(0.0136)	(0.0261)	(0.0353)	(0.0256)	(0.0247)
Partner Education: Secondary	0.0437^***^	0.0704^**^	0.0011	0.0251	0.0607^*^
	(0.0138)	(0.0287)	(0.0286)	(0.0247)	(0.0335)
Partner Education: Tetiary	0.1089^***^	0.1347^***^	0.0471^*^	0.1273^***^	0.0368
	(0.0166)	(0.0238)	(0.0280)	(0.0238)	(0.0516)
Muslim Dummy	−0.0216	0.0133	0.0078	−0.0649^***^	−0.0282
	(0.0159)	(0.0277)	(0.0204)	(0.0249)	(0.0280)
Ethnicity: Ga/Dangme	−0.0727^**^	−0.0029	0.0228	−0.1891^***^	
	(0.0327)	(0.0586)	(0.0262)	(0.0510)	
Ethnicity: Ewe and Guan	−0.0383^**^	−0.0496^**^	−0.0239	−0.0707^**^	−0.3705
	(0.0178)	(0.0243)	(0.0372)	(0.0318)	(0.3045)
Ethnicity: Northern Groups	0.0344^*^	−0.0098	0.0422^**^	0.0275	−0.1515^**^
	(0.0207)	(0.0416)	(0.0200)	(0.0239)	(0.0628)
Ethnicity: Others	−0.0184	−0.0014	0.0279	−0.0146	−0.5808^**^
	(0.0374)	(0.0608)	(0.0323)	(0.0633)	(0.2370)
Number of Elderly Women in HH	−0.0208^***^	−0.0089	−0.0174	−0.0082	−0.0288^**^
	(0.0074)	(0.0128)	(0.0125)	(0.0136)	(0.0131)
Wealth Quintile: Poorer	0.0483^***^	0.0544^**^	0.0280	0.0355^**^	0.0637^***^
	(0.0117)	(0.0211)	(0.0225)	(0.0181)	(0.0233)
Wealth Quintile: Middle	0.0493^***^	0.0712^***^	0.0321	0.0474^**^	0.0599
	(0.0133)	(0.0243)	(0.0279)	(0.0212)	(0.0389)
Wealth Quintile: Richer	0.1020^***^	0.1398^***^	0.0603	0.0884^***^	0.1322^***^
	(0.0131)	(0.0218)	(0.0384)	(0.0199)	(0.0322)
Wealth Quintile: Richest	0.1379^***^	0.1397^***^	0.1846^*^	0.1359^***^	0.0105
	(0.0142)	(0.0217)	(0.0953)	(0.0204)	(0.0769)
Eco_Zone: Greater Accra	−0.0466				
	(0.0331)				
Eco_Zone: Middle	0.0006				
	(0.0139)				
Eco_Zone: Northern	0.0475^**^				
	(0.0230)				
Rural Dummy	−0.0097	0.0147	−0.0224	0.0028	−0.0548
	(0.0155)	(0.0254)	(0.0519)	(0.0251)	(0.0366)
NSCPHGW	0.0718^***^	−0.0269	−0.0040	0.0334	0.1898^***^
	(0.0254)	(0.0380)	(0.0556)	(0.0268)	(0.0617)
NSCPHFT	0.0811^*^	0.2519^*^	−0.0254	0.1763^**^	0.0999
	(0.0486)	(0.1329)	(0.0352)	(0.0850)	(0.1574)
Year 2014	0.1569^***^	0.1974^***^	0.0978^**^	0.1862^***^	0.1212^**^
	(0.0195)	(0.0262)	(0.0386)	(0.0266)	(0.0476)
*N*	6386	1763	543	1922	2063
pseudo *R*^2^	0.168	0.180	0.322	0.182	0.153
p	0.0000	0.0000	0.0000	0.0000	0.0000

Source: Authors’ Calculations. Note: ^***^ is significant at *p* < 0.01, ^**^ is significant at p < 0.05, ^*^ is significant at *p* < 0.10. NSCPHGW, NSCPHFT are non-self cluster proportion of households with good water, non-self cluster proportion of households with flush toilet respectively. To account for women without Partners, a category was created for missing husbands under partner’s education but coefficient not reported

**Table 4:** Determinants of use of skilled birth attendants in Ghana – marginal effect estimates

Variables	Ecological Zones				
	National	Southern	Gt Accra	Middle	Northern
Woman’s_Age	0.0228^***^	0.0274^*^	0.0051	0.0293^**^	0.0002
	(0.0087)	(0.0160)	(0.0103)	(0.0143)	(0.0193)
Woman’s age Square	−0.0002^*^	−0.0003	−0.0000	−0.0003	0.0000
	(0.0001)	(0.0002)	(0.0002)	(0.0002)	(0.0003)
Birth Order: 2nd Birth	−0.1202^***^	−0.1098^**^	− 0.0488^*^	−0.1756^***^	−0.0899
	(0.0271)	(0.0487)	(0.0292)	(0.0519)	(0.0633)
Birth Order: 3rd Birth	−0.1828^***^	−0.1603^***^	−0.1066^*^	−0.2449^***^	−0.1285^**^
	(0.0329)	(0.0594)	(0.0551)	(0.0673)	(0.0573)
Birth Order: 4th Birth	−0.2313^***^	−0.2586^***^	−0.0876^*^	−0.2780^***^	−0.1579^**^
	(0.0311)	(0.0600)	(0.0477)	(0.0541)	(0.0704)
Woman’s Education: Primary	0.0517^***^	−0.0141	0.0058	0.0299	0.1649^***^
	(0.0189)	(0.0378)	(0.0133)	(0.0290)	(0.0458)
Woman’s Education: Secondary	0.1460^***^	0.1208^***^	0.0939^***^	0.1124^***^	0.1383^**^
	(0.0190)	(0.0334)	(0.0314)	(0.0309)	(0.0564)
Woman’s Education: Tetiary	0.2913^***^		0.0337^*^		
	(0.0284)		(0.0181)		
Partner Education: Primary	0.0804^***^	0.0033	−0.1007	0.0512	0.1602^***^
	(0.0206)	(0.0468)	(0.0847)	(0.0328)	(0.0443)
Partner Education: Secondary	0.0807^***^	0.0346	−0.0397^**^	0.0544	0.1464^***^
	(0.0208)	(0.0396)	(0.0173)	(0.0358)	(0.0399)
Partner Education: Tetiary	0.1617^***^	0.1422^**^	−0.0019	0.1432^***^	0.2039^**^
	(0.0294)	(0.0595)	(0.0412)	(0.0423)	(0.0895)
Muslim Dummy	−0.0829^***^	−0.0541	−0.0003	−0.0340	−0.1504^***^
	(0.0240)	(0.0369)	(0.0167)	(0.0351)	(0.0429)
Ethnicity: Ga/Dangme	−0.0323	0.0317	0.0386^***^	−0.1217^**^	
	(0.0449)	(0.0909)	(0.0137)	(0.0556)	
Ethnicity: Ewe and Guan	0.0036	0.0154	0.0136	−0.0619	−0.3282^***^
	(0.0310)	(0.0405)	(0.0161)	(0.0457)	(0.0540)
Ethnicity: Northern Groups	0.0568^*^	0.0440	0.0302^**^	0.0168	−0.4162^***^
	(0.0330)	(0.0652)	(0.0133)	(0.0351)	(0.0522)
Ethnicity: Others	0.0134	−0.0431	0.0075	0.0203	−0.3974^***^
	(0.0472)	(0.0854)	(0.0228)	(0.0632)	(0.0330)
Number of Elderly Women in HH	−0.0072	0.0171	0.0023	0.0278	−0.0444^*^
	(0.0112)	(0.0224)	(0.0041)	(0.0220)	(0.0252)
Wealth Quintile: Poorer	0.0145	0.0568	−0.0676	0.0110	0.0390
	(0.0218)	(0.0409)	(0.0454)	(0.0289)	(0.0508)
Wealth Quintile: Middle	0.0945^***^	0.1140^**^	−0.0195	0.1030^***^	0.2134^***^
	(0.0232)	(0.0460)	(0.0395)	(0.0308)	(0.0827)
Wealth Quintile: Richer	0.1745^***^	0.2395^***^	−0.0129	0.1582^***^	0.2645^***^
	(0.0239)	(0.0425)	(0.0329)	(0.0334)	(0.0963)
Wealth Quintile: Richest	0.2197^***^	0.1724^***^	0.0311	0.2039^***^	0.3937^***^
	(0.0277)	(0.0578)	(0.0390)	(0.0319)	(0.0895)
Eco_Zone: Greater Accra	0.0319				
	(0.0553)				
Eco_Zone: Middle	0.0754^***^				
	(0.0242)				
Eco_Zone: Northern	0.0019				
	(0.0388)				
Rural Dummy	−0.1455^***^	−0.1340^***^	−0.0719	−0.1032^***^	−0.2030^***^
	(0.0245)	(0.0386)	(0.0523)	(0.0371)	(0.0717)
NSCPHGW	0.1241^***^	−0.0679	0.0386	0.0903^**^	0.3738^***^
	(0.0355)	(0.0652)	(0.0464)	(0.0426)	(0.0933)
NSCPHFT	0.2588^***^	0.5073^***^	0.0633^*^	0.1851	0.3942
	(0.0891)	(0.1681)	(0.0339)	(0.1270)	(0.3705)
Year 2014	0.2874^***^	0.2757^***^	0.0523^**^	0.2194^***^	0.4062^***^
	(0.0242)	(0.0398)	(0.0227)	(0.0366)	(0.0428)
*N*	6444	1770	547	1944	2045
pseudo *R*^2^	0.284	0.198	0.456	0.232	0.317
p	0.0000	0.0000	0.0000	0.0000	0.0000

Source: Authors’ Calculations. Note: ^***^ is significant at p < 0.01, ^**^ is significant at p < 0.05, ^*^ is significant at p < 0.10. NSCPHGW, NSCPHFT are non-self cluster proportion of households with good water, non-self cluster proportion of households with flush toilet respectively. To account for women without Partners, a category was created for missing husbands under partner’s education but coefficient not reported

As earlier indicated, there are other aspects of the results in Tables 3 and 4 that deserves special mention. For example, the effect of women’s secondary and tertiary education on the two RHS is indifferent of the ecological zones. However, returns to primary education is important mostly in the resource poor ecological zones (southern and northern zones). Partners’ education follows the same pattern except that returns to partners primary education is significant (*p* < 0.05) only in the northern zone. Additionally, the effect of number of elderly women in a household on use of RHS is also significant mainly in the northern zone. The effect of NSCPHGW (a proxy for access to and availability of health services) is mainly significant in the middle and northern ecological zone for the two RHS. However, the other health service availability and accessibility proxy (NSCPHFT) was significant in the southern and middle zone in the case of 4+ antenatal visits, and the southern zone and Greater Accra in the case of skilled birth attendants. Finally, the results suggest that the probability of using the two reproductive health services increased in 2014 compared to 1998 in all the ecological zones. The northern and southern zones had the highest increase in the probability of using skilled birth attendants and 4+ antenatal visits respectively.

### Decomposition results

In this section, we present results of the decomposition as contained in Table 5. The top panel of the table shows the mean probability of use of the relevant reproductive health input for the two periods (Pr_0 = 2014) and (Pr_1 = 1998), the difference between the two probabilities (i.e. labeled “difference”) and the proportion of the difference explained by the determinants captured in the regression model (labeled “explained”). From Table 5, the level of increase in the probability of having 4+ antenatal visits between 1998 and 2014 is 0.232 (national), 0.253 (southern zone), 0.159 (Greater Accra), 0.241 (middle zone) and 0.214 (northern zone). The decomposition also suggest that the part of the difference explained by the determinants (i.e. from national to the ecological zones) is between 10% (southern zone) and 40% (northern zone). For delivery assistance, the increase in the average probability of use is 0.301 (national), 0.318 (southern zone), 0.179 (Greater Accra), 0.276 (middle zone) and 0.377 (northern zone).

**Table 5:** A decomposition of changes in 4+ antenatal visits and use of skilled attendants during delivery in Ghana between 1998 and 2014

Determinants	4+ Antenatal Visits					Skilled Delivery Assistance				
	National	Southern	Gt. Accra	Middle	Northern	National	Southern	Gt. Accra	Middle	Northern
Pr_0	0.8771	0.8766	0.9139	0.8976	0.8065	0.7570	0.7352	0.9318	0.8106	0.5355
Pr_1	0.6451	0.6238	0.7544	0.6562	0.5921	0.4565	0.4168	0.7529	0.5350	0.1586
Difference	0.2319	0.2527	0.1596	0.2414	0.2144	0.3006	0.3184	0.1790	0.2756	0.3769
Explained	0.0461	0.0242	0.0224	0.0474	0.0863	0.0991	0.0342	0.1857	0.1243	0.1332
Contributions										
Woman’s Age	0.0042	0.0066	0.0118	0.0073	−0.0008	0.0029	0.0054	0.0036	0.0115^**^	−0.0018
	(0.0030)	(0.0059)	(0.0105)	(0.0057)	(0.0032)	(0.0018)	(0.0043)	(0.0069)	(0.0057)	(0.0019)
%	9.1	27.3	52.7	15.4	−0.9	2.9	15.8	1.9	9.3	−1.4
Birth Order	0.0008	0.0009	0.0205	−0.0009	0.0008	0.0012	0.0027	−0.0033	−0.0044	−0.0004
	(0.0027)	(0.0049)	(0.0130)	(0.0052)	(0.0034)	(0.0020)	(0.0040)	(0.0090)	(0.0055)	(0.0025)
%	1.7	3.7	91.5	−1.9	0.9	1.2	7.9	−1.8	−3.5	−0.3
Woman’s Education	0.0062^***^	0.0078^*^	−0.0060	0.0024	0.0124^***^	0.0119^***^	0.0149^***^	0.0209	0.0036	0.0153^***^
	(0.0022)	(0.0043)	(0.0136)	(0.0033)	(0.0043)	(0.0028)	(0.0056)	(0.0138)	(0.0031)	(0.0056)
%	13.4	32.2	−26.8	5.1	14.4	12.0	43.6	11.3	2.9	11.5
Partner’s Education	−0.0006	−0.0037	0.0190^*^	−0.0006	0.0058	0.0030	−0.0030	−0.0022	0.0088^**^	0.0161^***^
	(0.0022)	(0.0042)	(0.0104)	(0.0039)	(0.0046)	(0.0025)	(0.0048)	(0.0088)	(0.0044)	(0.0058)
%	−1.3	−15.3	84.8	−1.3	6.7	3.0	−8.8	−1.2	7.1	12.1
Ethnicity	0.0039^*^	−0.0007	−0.0142	0.0039	0.0005	0.0051^***^	0.0015	−0.0012	0.0064**	0.0004
	(0.0022)	(0.0023)	(0.0094)	(0.0029)	(0.0025)	(0.0019)	(0.0027)	(0.0110)	(0.0030)	(0.0026)
%	8.5	−2.9	−63.4	8.2	0.6	5.1	4.4	−0.6	5.1	0.3
Muslim Dummy	−0.0002	−0.0029	−0.0034	−0.0015	−0.0036	0.0007	−0.0005	−0.0078	−0.0005	0.0084^***^
	(0.0006)	(0.0022)	(0.0058)	(0.0016)	(0.0038)	(0.0006)	(0.0032)	(0.0093)	(0.0010)	(0.0032)
%	−0.4	−12.0	−15.2	−3.2	−4.2	0.7	−1.5	−4.2	−0.4	6.3
No. of Elderly Women	0.0000	0.0003	−0.0024	0.0002	−0.0041^***^	0.0000	−0.0045^**^	0.0068	−0.0001	−0.0021^*^
	(0.0003)	(0.0016)	(0.0034)	(0.0011)	(0.0014)	(0.0002)	(0.0022)	(0.0115)	(0.0012)	(0.0011)
%	0	1.2	−10.7	0.4	−4.8	0.0	−13.2	3.7	−0.1	−1.6
Household Wealth	0.0074^***^	0.0207^***^	0.0117	0.0049	0.0002	0.0116^***^	0.0209^***^	−0.0028	0.0130^***^	−0.0050
	(0.0025)	(0.0070)	(0.0152)	(0.0030)	(0.0050)	(0.0020)	(0.0073)	(0.0077)	(0.0041)	(0.0044)
%	16.1	85.5	52.2	10.3	0.2	11.7	61.1	−1.5	10.5	−3.8
Eco Zones	−0.0026					−0.0012				
	(0.0027)					(0.0020)				
%	−5.6					−1.2				
Water and Sanitation	0.0298^***^	0.0037	−0.0110	0.0259^**^	0.0724^***^	0.0486^***^	−0.0114	0.1693^***^	0.0654^***^	0.0945^***^
	(0.0082)	(0.0116)	(0.0398)	(0.0128)	(0.0141)	(0.0088)	(0.0132)	(0.0278)	(0.0162)	(0.0122)
%	64.6	15.3	−49.1	54.6	83.9	49.0	−33.3	91.2	52.6	70.9
Rural Dummy	−0.0028	−0.0086^*^	−0.0036	0.0058	0.0028	0.0152^***^	0.0083^*^	0.0027	0.0208^***^	0.0081
	(0.0036)	(0.0045)	(0.0076)	(0.0068)	(0.0046)	(0.0038)	(0.0045)	(0.0069)	(0.0073)	(0.0050)
%	−6.1	−35.5	−16.1	12.2	3.2	15.3	24.3	1.5	16.7	6.1
*Observations*	6386	1809	543	1969	2065	6444	1816	547	1992	2089

Source: Authors’ Calculations. Note: ^***^ is significant at p < 0.01, ^**^ is significant at p < 0.05, ^*^ is significant at p < 0.10. Contribution estimates are mean values of the decomposition, bootstrapped to 5000 replications. Note that the base group (Pr = 0) is 1998 and the reference group (Pr = 1) is 2014. Note also that the contribution of the error term has been ignored in the current estimates as is common in the literature

The second panel of the decomposition results shows individual contributions of the determinants. A positive percentage contribution is indicative that the determinant in question contributed to increasing the probability of use and vice versa for a negative value. The results in Table 5 suggest that the contribution of the woman’s age was only significant in the case of skilled delivery for the middle belt. Per the results, women’s education constitutes a key variable contributing to the increased probability of using the two RHS. This is because except for Greater Accra and the middle belt, the women’s education coefficients are significant across geography and the two RHS. Most importantly, the percentage contribution is highest for both 4+ antenatal visits and skilled attendants at birth in the southern (32.2% and 43.6%) and northern (14.4% and 11.5%) zones respectively.

Additionally, partner’s education is significant, contributing about 84.8% to the increased probability of having 4+ antenatal visits in Greater Accra and 7.1% and 12.1% respectively of the increased probability of using skilled birth services in the middle and northern zones. Ethnicity is also significant at the national level and in the middle zone but contributing only a marginal 5.1% in each case to the increased probability of using skilled birth attendants. Being a Muslim is only significant in the northern belt with respect to skilled birth attendants but contributing a marginal 6.3% to the period increase in the probability of using skilled birth attendants. The number of elderly women in a household reduces the usage gap, with the estimates being significant for 4+ antenatal visit in the northern zone (4.8%), skilled birth attendants in the southern (13.2%) and northern zones (1.6%). Household wealth is also important and significant at the national level. However, it most important effect is in the southern zone, where it contributed 86% and 61% respectively to the increased probability of women making 4+ antenatal visits and using skilled birth attendant during childbirth.

The results in Table 5 shows that the contribution of the health facility availability and accessibility proxies to the increased probability (between 1998 and 2014) of a woman having either 4+ antenatal visits or skilled birth attendants is significant at the national level and the middle and southern zones. At the national level, availability and accessibility contributes about 65% and 49% respectively to the increased probability of use of 4+ antenatal visit and skilled birth attendants. At the ecological zone level, availability and accessibility of health services contributed 54.6% (middle zone) and 85% (northern zone) to the change in 4+ antenatal visits and 91% (Greater Accra), 53% (middle zone) and 71% (northern zone) to the change in the use of skilled birth attendants. As expected, location of residence is a statistically significant contributor to changes in the use of skilled birth attendants between 1998 and 2014. What is however surprising is the fact that living in a rural area significantly contributed positively (i.e. national level: 15%, southern belt: 24% and middle belt: 17%) to the change in the use of skilled birth attendants between 1998 and 2014.

## Discussion

Overall the findings of the study suggest that utilization of RHS improved between 1998 and 2014 both at the national and ecological zone levels. From the results, factors such as women’s age, being a Muslim, number of elderly women in the household and living in a rural area are all significantly negatively correlated with utlisation of RHS, Also, women and partners education, household wealth and access to and availability of health services are significantly positively correlated utilization of the two RHS. Additionally, women and partners education, household wealth and availability and accessibility to health facilities are key factors influencing changes in utilization of RHS services both at the national and ecological zone levels. Although the northern and southern belts have lower levels of women and partner education and availability and accessibility of health facilities, they nonetheless recorded the greatest improvements both in education and utilization of the two RHS. Thus, the significant contribution of women and partners education and availability and accessibility of health facilities to the increased probability of using the two RHS could be attributed to the marginal efficiency of educational and health infrastructure investments in the two ecological zones. In other words, educational and health infrastructure investments in the northern and southern zones generate higher marginal returns compared to Greater Accra and the middle zone where levels of education and health infrastructure are already high.

The results of the effect of individual, household and community level factors on the use of the two RHS is consistent with the existing literature. For example, women’s age has been found to have a quadratic effect on the utilization of reproductive health services [7–10]. Women’s education has also been discussed in the RHS literature as a key determinant of use of RHS. It is often argued that the correlation between education and use of health services come from autonomy [27–29] and health production efficiency [30, 31]. Female education in particular is argued to improve women’s autonomy through the development of capabilities and confidence that helps women to make decisions about their own health. There is evidence in the bargaining literature that suggests that male education enhances female autonomy and empowerment.

Existing evidence from Ghana [9, 10], Rwanda, Uganda, Turkey, Bangladesh and Thailand [6, 13, 32–34] suggest that household wealth is a key determinant of utilization of RHS. Consistent with the results, prior studies have found a significant positive correlation between household wealth and utilization of RHS. However, given that RHS such as antenatal and delivery care are supposed to be available and free in all public hospitals in Ghana, the relative importance of household wealth may be a confirmation of the argument that the indirect cost of reproductive healthcare (i.e. travel cost and opportunity cost) may be so high in Ghana [10] that free direct prices may not constitute enough incentives for pregnant women to use RHS. The positive correlation between the proxies for accessibility and availability of health services is also consistent with the existing literature. Different measures of health services availability and accessibility such as non-self cluster proportion of health utilisations variables [10, 20], Distance from health facility [7], constraints to seeking care [9] have all been found to be positively correlated with utilization of health services. Consistent with the results, other factors such as ethnicity [35, 36], religion [7–9, 16] and household size [9, 10] have also been argued in the RHS literature as correlates of utilization of RHS.

With respect to the decomposition results, the existing evidence [11–13] suggest that health behavior, education, responsive health systems and availability and accessibility of health services have been key drivers of change in utilization of health services in countries like Ghana, Rwanda and Uganda. The results of the paper emphasizes this fact and more so in the northern zone. The importance of availability and accessibility to health services in the northern zone is due to the fact that it has for some time now been the target of several donors and NGOs for the implementation of several RHS interventions and research. Thus, the progress suggested by the results may well be related to the outcomes of the several studies and RHS interventions on-going in the northern zones. Related to the donor and NGO effect is the fact that the northern zone has benefited immensely from the Ministry of Health’s policy to bridge the health outcomes gap between the southern and the northern parts of Ghana. A major intervention in this direction has been the provision of Community Health Planning and Services (CHPS) compounds aimed at improving access to health services especially in the rural and remote parts of the country.^1^ For example data from the District Health Information Systems (DHIMS) of the Ghana Health Service (GHS) suggest that about 722 CHPS compounds have been put up in the northern zone.

It is important though to empahsise that the distribution of CHPS facilities in the northern zone is not uniform but skewed (i.e. a higher concentration in Upper East followed by Upper West and the Northern region as shown in Figs. 1 and 2). It is also important to note that other zones such as the middle belt have benefited from the CHPS programme more than the northern and southern zones (see Fig. 1) and yet did not record the kind of improvements in the use of the two RCH as seen in the northern and southern zones. Thus, the recorded improvements in the northern and southern zones may be related to the efficiency of the existing stock of health infrastructure (i.e. marginal efficiency of investments) and not just the numbers.

## Policy implications

The results of the study as discussed above, are straightforward and unambiguous. However, there are a couple of issues that needs further emphasis for the purposes of policy development and interventions targeting.

The fact that the resource-poor zones (i.e. southern and northern) recorded the highest levels of improvements in 4+ antenatal visits and skilled birth attendants respectively is instructive. This may be an indication that pro-poor policies implemented by the health sector is yielding results. Thus, there may be the need to scale-up such interventions in order to speed up the rate of progress. Also important is the fact that the two resource-poor zones that seem to have witnessed the greatest improvements in both 4+ antenatal visits and skilled birth attendance also had the greatest improvements in both women and partners education, household wealth and availability and accessibility to health facilities. As it turned out, and also suggested by the decomposition results, these same factors were key in explaining the positive change in the probability of using the two reproductive health services especially in the two resource-poor zones. Even more important is the fact that the marginal efficiency of educational investments was higher in the two resource-poor zones (southern and northern) compared to the others. Given also that primary and secondary education tend to be very important in terms of education’s effect on the use of reproductive health services (as per the regression results), policy-makers may have to prioritise and invest more in primary and secondary education, especially in the two resource poor zones. Such investment as the results seem to suggest, may be crucial to further improving reproductive health outcomes in resource-poor regions in Ghana.

## Limitations of the study

It is important to emphasise that the study would have benefited from expanding the scope of outcome variables and countries used. This would have made it possible to generalise the results for RHS in general. Secondly, the cross-sectional nature of the data and the absence of direct measures of health services availability and accessibility could introduce some form of biases in the results. This not withstanding, the data used is nationally representative and the econometric model robust. Above all, the results are consistent with the existing literature.

## Conclusion

The results make it clear that resource poor zones in Ghana such as the northern and southern zones lag behind the middle belt and Greater Accra in terms of utilisation of RHS. This not withstanding, improvements in primary and secondary education as well as health related investments have been key to appreciable improvements in the utilisation of RHS from 1998 to 2014. There is therefore the need for policy makers to scale-up investments in existing pro-poor interventions, especially in the area of education (i.e. primary and secondary) and health to improve the levels of utilisation of RHS in general and especially in resource poor zones such as the southern and northern zones in Ghana.

## Additional file

Additional file 1: **Table S1.** Determinants of 4+ Antenatal Visits in Ghana – Log Odds with Confidence Intervals. Contains regression estimates of the determinants of 4+ antenatal visits in Ghana, with the coefficients expressed in log odds and also showing the confidence intervals. **Table S2.** Determinants Use of Skilled Birth Attendants in Ghana – Log Odds with Confidence Intervals. Contains regression estimates of use of skilled birth attendants in Ghana, with the coefficients expressed in log odds and also showing the confidence intervals. (DOCX 38 kb)

## Footnotes

1 CHPS are lower level health facilities put up and operated by the staff of the Ministry of Health and normally found in the rural areas. The basic idea behind CHPS is enable rural dwellers to have easy access to health facilities and more importantly, reproductive health services for pregnant mothers.
