# Supplementary material for: Spatial variation in the use of reproductive health services over time: a decomposition analysis
Source: BMC Pregnancy Childbirth. 2018 Mar 6;18:63. doi: 10.1186/s12884-018-1695-3 (PMC5838884; doi:10.1186/s12884-018-1695-3)
Supplement: Supplementary file 1 — Table S1. Determinants of 4+ Antenatal Visits in Ghana – Log Odds with Confidence Intervals. Contains regression estimates of the determinants of 4+ antenatal visits in Ghana, with the coefficients expressed in log odds and also showing the confidence intervals. Table S2. Determinants Use of Skilled Birth Attendants in Ghana – Log Odds with Confidence Intervals. Contains regression estimates of use of skilled birth attendants in Ghana, with the coefficients expressed in log odds and also showing the confidence intervals. (DOCX 38 kb) [file 12884_2018_1695_MOESM1_ESM.docx]

**Additional file**

**Table S1: Determinants of 4+ Antenatal Visits in Ghana – Log Odds with Confidence Intervals**

|  | **Ecological Zones** | | | | |
| --- | --- | --- | --- | --- | --- |
| **Variables** | **National** | **Southern** | **Gt Accra** | **Middle** | **Northern** |
| Woman’s_Age | 0.1728*** | 0.1928** | 0.3733** | 0.2058*** | 0.0404 |
|  | (0.0410) | (0.0832) | (0.1763) | (0.0755) | (0.0670) |
|  | 0.09, 0.25 | 0.03, 0.36 | 0.03, 0.72 | 0.06, 0.35 | -0.09, 0.17 |
| Woman’s age Square | -0.0021*** | -0.0022* | -0.0054* | -0.0026** | -0.0005 |
|  | (0.0006) | (0.0012) | (0.0028) | (0.0011) | (0.0009) |
|  | -0.00, -0.00 | -0.00, 0.00 | -0.01, 0.00 | -0.00, -0.00 | -0.00, 0.00 |
| Birth Order: 2^nd^ Birth | -0.3060** | -0.3662 | -1.0750** | -0.4803* | 0.1434 |
|  | (0.1298) | (0.2276) | (0.4551) | (0.2452) | (0.2508) |
|  | -0.56, -0.05 | -0.81, 0.08 | -1.97, -0.18 | -0.96, 0.00 | -0.35, 0.64 |
| Birth Order: 3^rd^ Birth | -0.4032*** | -0.4304 | -0.9367* | -0.6876** | 0.0519 |
|  | (0.1461) | (0.2759) | (0.5147) | (0.2897) | (0.2612) |
|  | -0.69, -0.12 | -0.97, 0.11 | -1.95, 0.07 | -1.26, -0.12 | -0.46, 0.56 |
| Birth Order: 4^th^ Birth | -0.7034*** | -0.8040*** | -0.8776 | -0.9427*** | -0.1570 |
|  | (0.1515) | (0.2966) | (0.5383) | (0.2996) | (0.2737) |
|  | -1.00, -0.41 | -1.39, -0.22 | -1.93, 0.18 | -1.53, -0.36 | -0.69, 0.38 |
| Woman’s Education: Primary | 0.2535** | 0.3024* | 0.2609 | -0.1089 | 0.5807*** |
|  | (0.0999) | (0.1708) | (0.3610) | (0.1776) | (0.2088) |
|  | 0.06, 0.45 | -0.03, 0.64 | -0.45, 0.97 | -0.46, 0.24 | 0.17, 0.99 |
| Woman’s Education: Secondary | 0.6498*** | 0.8023*** | 1.6022*** | 0.2283 | 0.5245* |
|  | (0.1145) | (0.1811) | (0.3879) | (0.1959) | (0.2832) |
|  | 0.43, 0.87 | 0.45, 1.16 | 0.84, 2.36 | -0.16, 0.61 | -0.03, 1.08 |
| Woman’s Education: Tetiary | 1.4248* | . | 1.8113 | . | 1.3449 |
|  | (0.7628) | . | (1.2093) | . | (1.2210) |
|  | -0.07, 2.92 | **-** | -0.56, 4.18 | **-** | -1.05, 3.74 |
| Partner Education: Primary | 0.2814** | 0.2148 | -0.2112 | 0.1772 | 0.5293** |
|  | (0.1222) | (0.2176) | (0.5176) | (0.2389) | (0.2097) |
|  | 0.04, 0.52 | -0.21, 0.64 | -1.23, 0.80 | -0.29, 0.65 | 0.12, 0.94 |
| Partner Education: Secondary | 0.3412*** | 0.5023** | 0.0185 | 0.2056 | 0.4448 |
|  | (0.1071) | (0.1970) | (0.4916) | (0.1991) | (0.2711) |
|  | 0.13, 0.55 | 0.12, 0.89 | -0.95, 0.98 | -0.18, 0.60 | -0.09, 0.98 |
| Partner Education: Tetiary | 1.2079*** | 1.5979*** | 1.1602 | 1.9356** | 0.2628 |
|  | (0.2768) | (0.5016) | (0.9917) | (0.7977) | (0.3958) |
|  | 0.67, 1.75 | 0.61, 2.58 | -0.78, 3.10 | 0.37, 3.50 | -0.51, 1.04 |
| Muslim Dummy | -0.1633 | 0.1004 | 0.1395 | -0.4844*** | -0.1888 |
|  | (0.1182) | (0.2152) | (0.3751) | (0.1674) | (0.1901) |
|  | -0.39, 0.07 | -0.32, 0.52 | -0.60, 0.87 | -0.81, -0.16 | -0.56, 0.18 |
| Ethnicity: Ga/Dangme | -0.4858** | -0.0215 | 0.4181 | -1.1391*** | . |
|  | (0.1919) | (0.4271) | (0.5303) | (0.2495) | . |
|  | -0.86, -0.11 | -0.86, 0.82 | -0.62, 1.46 | -1.63, -0.65 | **-** |
| Ethnicity: Ewe and Guan | -0.2764** | -0.3484** | -0.3711 | -0.5102** | -1.7259 |
|  | (0.1210) | (0.1611) | (0.5094) | (0.2032) | (1.2505) |
|  | -0.51, -0.04 | -0.66, -0.03 | -1.37, 0.63 | -0.91, -0.11 | -4.18, 0.72 |
| Ethnicity: Northern Groups | 0.2714 | -0.0708 | 1.0419 | 0.2426 | -1.6121 |
|  | (0.1673) | (0.2954) | (0.6745) | (0.2239) | (1.2241) |
|  | -0.06, 0.60 | -0.65, 0.51 | -0.28, 2.36 | -0.20, 0.68 | -4.01, 0.79 |
| Ethnicity: Others | -0.1358 | -0.0101 | 0.6103 | -0.1171 | -2.6732** |
|  | (0.2646) | (0.4462) | (0.9519) | (0.4884) | (1.3066) |
|  | -0.65, 0.38 | -0.88, 0.86 | -1.26, 2.48 | -1.07, 0.84 | -5.23, -0.11 |
| Number of Elderly Women in HH | -0.1610*** | -0.0657 | -0.3002 | -0.0688 | -0.1908** |
|  | (0.0555) | (0.0940) | (0.2207) | (0.1132) | (0.0819) |
|  | -0.27, -0.05 | -0.25, 0.12 | -0.73, 0.13 | -0.29, 0.15 | -0.35, -0.03 |
| Wealth Quintile: Poorer | 0.4049*** | 0.4274** | 0.6070 | 0.3122* | 0.4684*** |
|  | (0.1014) | (0.1736) | (0.5888) | (0.1655) | (0.1811) |
|  | 0.21, 0.60 | 0.09, 0.77 | -0.55, 1.76 | -0.01, 0.64 | 0.11, 0.82 |
| Wealth Quintile: Middle | 0.4176*** | 0.5683*** | 0.7013 | 0.4302** | 0.4494 |
|  | (0.1212) | (0.2010) | (0.7588) | (0.2088) | (0.3275) |
|  | 0.18, 0.66 | 0.17, 0.96 | -0.79, 2.19 | 0.02, 0.84 | -0.19, 1.09 |
| Wealth Quintile: Richer | 0.9980*** | 1.3729*** | 1.2788 | 0.8977*** | 1.2494*** |
|  | (0.1585) | (0.2640) | (0.9232) | (0.2403) | (0.4384) |
|  | 0.69, 1.31 | 0.86, 1.89 | -0.53, 3.09 | 0.43, 1.37 | 0.39, 2.11 |
| Wealth Quintile: Richest | 1.5868*** | 1.6301*** | 2.5534*** | 1.7579*** | 0.0708 |
|  | (0.2523) | (0.4587) | (0.9877) | (0.3894) | (0.5317) |
|  | 1.09, 2.08 | 0.73, 2.53 | 0.62, 4.49 | 0.99, 2.52 | -0.97, 1.11 |
| Eco_Zone: Greater Accra | -0.3286 | . | . | . | . |
|  | (0.2140) | . | . | . | . |
|  | -0.75, 0.09 |  |  |  |  |
| Eco_Zone: Middle | 0.0046 | . | . | . | . |
|  | (0.1074) | . | . | . | . |
|  | -0.21, 0.22 | **-** | **-** | **-** | **-** |
| Eco_Zone: Northern | 0.3849* | . | . | . | . |
|  | (0.1976) | . | . | . | . |
|  | -0.00, 0.77 | **-** | **-** | **-** | **-** |
| Rural Dummy | -0.0753 | 0.1072 | -0.3497 | 0.0232 | -0.3880 |
|  | (0.1208) | (0.1835) | (0.7364) | (0.2085) | (0.2707) |
|  | -0.31, 0.16 | -0.25, 0.47 | -1.79, 1.09 | -0.39, 0.43 | -0.92, 0.14 |
| NSCPHGW | 0.5549*** | -0.1988 | -0.0694 | 0.2793 | 1.2576*** |
|  | (0.1930) | (0.2791) | (0.9584) | (0.2230) | (0.4004) |
|  | 0.18, 0.93 | -0.75, 0.35 | -1.95, 1.81 | -0.16, 0.72 | 0.47, 2.04 |
| NSCPHFT | 0.6270* | 1.8606* | -0.4380 | 1.4725** | 0.6620 |
|  | (0.3766) | (1.0212) | (0.6101) | (0.7239) | (1.0403) |
|  | -0.11, 1.37 | -0.14, 3.86 | -1.63, 0.76 | 0.05, 2.89 | -1.38, 2.70 |
| Year 2014 | 1.0819*** | 1.2876*** | 1.3775*** | 1.3640*** | 0.7430*** |
|  | (0.1313) | (0.1589) | (0.4646) | (0.1871) | (0.2822) |
|  | 0.82, 1.34 | 0.98, 1.60 | 0.47, 2.29 | 1.00, 1.73 | 0.19, 1.30 |
| Constant | -3.1450*** | -3.9501*** | -6.1453** | -3.1361*** | 1.2305 |
|  | (0.6599) | (1.2957) | (2.8783) | (1.1253) | (1.7609) |
|  | -4.44, -1.85 | -6.49, -1.41 | -11.79, -0.5 | -5.34, -0.93 | -2.22, 4.68 |
| *N* | 6386 | 1763 | 543 | 1922 | 2063 |
| pseudo *R*^2^ | 0.168 | 0.180 | 0.322 | 0.182 | 0.153 |
| p | 0.0000 | 0.0000 | 0.0000 | 0.0000 | 0.0000 |

Source: Authors’ Calculations. Note: *** is significant at p<0.01, ** is significant at p<0.05, * is significant at p<0.10.

Figures in parentheses are standard errors with those not in parentheses but following the standard errors being confidence

Intervals. NSCPHGW, NSCPHFT are non-self cluster proportion of households with good water, non-self cluster proportion of households with flush toilet respectively. To account for women without Partners, a category was created for missing

husbands under partner’s education but coefficient not reported.

**Table S2: Determinants Use of Skilled Birth Attendants in Ghana – Log Odds with Confidence Intervals**

|  | **Ecological Zones** | | | | |
| --- | --- | --- | --- | --- | --- |
| **Variables** | **National** | **Southern** | **Gt Accra** | **Middle** | **Northern** |
| Woman’s_Age | 0.1089*** | 0.1218* | 0.1392 | 0.1640** | 0.0009 |
|  | (0.0414) | (0.0715) | (0.2695) | (0.0799) | (0.0786) |
|  | 0.03, 0.19 | -0.02, 0.26 | -0.39, 0.67 | 0.01, 0.32 | -0.15, 0.15 |
| Woman’s age Square | -0.0012* | -0.0012 | -0.0012 | -0.0018 | 0.0001 |
|  | (0.0006) | (0.0011) | (0.0044) | (0.0012) | (0.0011) |
|  | -0.00, 0.00 | -0.00, 0.00 | -0.01, 0.01 | -0.00, 0.00 | -0.00, 0.00 |
| Birth Order: 2^nd^ Birth | -0.5427*** | -0.4701** | -1.0111** | -0.8715*** | -0.3740 |
|  | (0.1168) | (0.2035) | (0.4060) | (0.2365) | (0.2715) |
|  | -0.77, -0.31 | -0.87, -0.07 | -1.81, -0.22 | -1.34, -0.41 | -0.91, 0.16 |
| Birth Order: 3^rd^ Birth | -0.8034*** | -0.6756*** | -1.7211*** | -1.1603*** | -0.5423** |
|  | (0.1357) | (0.2436) | (0.4658) | (0.2865) | (0.2565) |
|  | -1.07, -0.54 | -1.15, -0.20 | -2.63, -0.81 | -1.72, -0.60 | -1.05, -0.04 |
| Birth Order: 4^th^ Birth | -1.0896*** | -1.1391*** | -1.5765*** | -1.4934*** | -0.6469** |
|  | (0.1454) | (0.2706) | (0.5499) | (0.2814) | (0.2939) |
|  | -1.37, -0.80 | -1.67, -0.61 | -2.65, -0.50 | -2.04, -0.94 | -1.22, -0.07 |
| Woman’s Education: Primary | 0.2555*** | -0.0623 | 0.1643 | 0.1717 | 0.6658*** |
|  | (0.0962) | (0.1660) | (0.3953) | (0.1707) | (0.1882) |
|  | 0.07, 0.44 | -0.39, 0.26 | -0.61, 0.94 | -0.16, 0.51 | 0.30, 1.03 |
| Woman’s Education: Secondary | 0.7220*** | 0.5381*** | 1.9779*** | 0.6176*** | 0.5573** |
|  | (0.0974) | (0.1513) | (0.4320) | (0.1659) | (0.2286) |
|  | 0.53, 0.91 | 0.24, 0.83 | 1.13, 2.82 | 0.29, 0.94 | 0.11, 1.01 |
| Woman’s Education: Tetiary | 2.9091*** | . | 1.5072 | . | . |
|  | (1.0886) | . | (1.0735) | . | . |
|  | 0.78, 5.04 | **-** | -0.60, 3.61 | - | - |
| Partner Education: Primary | 0.4139*** | 0.0145 | -1.4559* | 0.3061 | 0.6466*** |
|  | (0.1149) | (0.2087) | (0.7648) | (0.2098) | (0.1816) |
|  | 0.19, 0.64 | -0.39, 0.42 | -2.95, 0.04 | -0.11, 0.72 | 0.29, 1.00 |
| Partner Education: Secondary | 0.3888*** | 0.1528 | -1.2177** | 0.2985 | 0.5903*** |
|  | (0.1004) | (0.1746) | (0.5537) | (0.1927) | (0.1628) |
|  | 0.19, 0.59 | -0.19, 0.49 | -2.30, -0.13 | -0.08, 0.68 | 0.27, 0.91 |
| Partner Education: Tetiary | 0.9392*** | 0.7135** | -0.0518 | 1.0567** | 0.8305** |
|  | (0.2159) | (0.3478) | (1.0769) | (0.4422) | (0.3841) |
|  | 0.52, 1.36 | 0.03, 1.40 | -2.16, 2.06 | 0.19, 1.92 | 0.08, 1.58 |
| Muslim Dummy | -0.3853*** | -0.2349 | -0.0090 | -0.1843 | -0.6125*** |
|  | (0.1084) | (0.1568) | (0.4516) | (0.1854) | (0.1776) |
|  | -0.60, -0.17 | -0.54, 0.07 | -0.89, 0.88 | -0.55, 0.18 | -0.96, -0.26 |
| Ethnicity: Ga/Dangme | -0.1506 | 0.1440 | 1.2170** | -0.6010** | . |
|  | (0.2045) | (0.4231) | (0.4774) | (0.2493) | . |
|  | -0.55, 0.25 | -0.69, 0.97 | 0.28, 2.15 | -1.09, -0.11 | - |
| Ethnicity: Ewe and Guan | 0.0171 | 0.0688 | 0.4136 | -0.3237 | -1.7758*** |
|  | (0.1489) | (0.1815) | (0.5436) | (0.2267) | (0.4748) |
|  | -0.27, 0.31 | -0.29, 0.42 | -0.65, 1.48 | -0.77, 0.12 | -2.71, -0.85 |
| Ethnicity: Northern Groups | 0.2751* | 0.2008 | 1.2759 | 0.0954 | -1.9297*** |
|  | (0.1619) | (0.3078) | (0.8261) | (0.2026) | (0.3251) |
|  | -0.04, 0.59 | -0.40, 0.80 | -0.34, 2.90 | -0.30, 0.49 | -2.57, -1.29 |
| Ethnicity: Others | 0.0648 | -0.1865 | 0.2226 | 0.1171 | -2.7625*** |
|  | (0.2313) | (0.3615) | (0.7377) | (0.3758) | (0.6027) |
|  | -0.39, 0.52 | -0.90, 0.52 | -1.22, 1.67 | -0.62, 0.85 | -3.94, -1.58 |
| Number of Elderly Women in HH | -0.0345 | 0.0761 | 0.0625 | 0.1552 | -0.1803* |
|  | (0.0532) | (0.0997) | (0.1131) | (0.1226) | (0.1031) |
|  | -0.14, 0.07 | -0.12, 0.27 | -0.16, 0.28 | -0.09, 0.40 | -0.38, 0.02 |
| Wealth Quintile: Poorer | 0.0701 | 0.2568 | -1.1291** | 0.0620 | 0.1577 |
|  | (0.1061) | (0.1894) | (0.4584) | (0.1641) | (0.2045) |
|  | -0.14, 0.28 | -0.11, 0.63 | -2.03, -0.23 | -0.26, 0.38 | -0.24, 0.56 |
| Wealth Quintile: Middle | 0.4838*** | 0.5269** | -0.4457 | 0.6338*** | 0.8700** |
|  | (0.1273) | (0.2235) | (0.7506) | (0.2075) | (0.3558) |
|  | 0.23, 0.73 | 0.09, 0.96 | -1.92, 1.03 | 0.23, 1.04 | 0.17, 1.57 |
| Wealth Quintile: Richer | 0.9799*** | 1.2589*** | -0.3265 | 1.0679*** | 1.0970** |
|  | (0.1610) | (0.2808) | (0.7443) | (0.2729) | (0.4419) |
|  | 0.66, 1.30 | 0.71, 1.81 | -1.79, 1.13 | 0.53, 1.60 | 0.23, 1.96 |
| Wealth Quintile: Richest | 1.3468*** | 0.8901** | 0.8058 | 1.6054*** | 1.8139*** |
|  | (0.2297) | (0.3638) | (1.0099) | (0.3689) | (0.5868) |
|  | 0.90, 1.80 | 0.18, 1.60 | -1.17, 2.79 | 0.88, 2.33 | 0.66, 2.96 |
| Eco_Zone: Greater Accra | 0.1565 | . | . | . | . |
|  | (0.2797) | . | . | . | . |
|  | -0.39, 0.70 | **-** | **-** | **-** | **-** |
| Eco_Zone: Middle | 0.3718*** | . | . | . | . |
|  | (0.1228) | . | . | . | . |
|  | 0.13, 0.61 | 0.13, 0.61 | 0.13, 0.61 | 0.13, 0.61 | 0.13, 0.61 |
| Eco_Zone: Northern | 0.0089 | . | . | . | . |
|  | (0.1857) | . | . | . | . |
|  | -0.36, 0.37 | **-** | **-** | **-** | **-** |
| Rural Dummy | -0.7321*** | -0.6244*** | -1.2883* | -0.6020*** | -0.8234*** |
|  | (0.1270) | (0.1872) | (0.7057) | (0.2255) | (0.2987) |
|  | -0.98, -0.48 | -0.99, -0.26 | -2.67, 0.09 | -1.04, -0.16 | -1.41, -0.24 |
| NSCPHGW | 0.5935*** | -0.3013 | 1.0460 | 0.5046** | 1.5196*** |
|  | (0.1680) | (0.2901) | (1.1165) | (0.2379) | (0.3799) |
|  | 0.26, 0.92 | -0.87, 0.27 | -1.14, 3.23 | 0.04, 0.97 | 0.78, 2.26 |
| NSCPHFT | 1.2373*** | 2.2522*** | 1.7166** | 1.0347 | 1.6026 |
|  | (0.4313) | (0.7499) | (0.8280) | (0.7164) | (1.5050) |
|  | 0.39, 2.08 | 0.78, 3.72 | 0.09, 3.34 | -0.37, 2.44 | -1.35, 4.55 |
| Year 2014 | 1.3135*** | 1.1972*** | 1.1812** | 1.1530*** | 1.8505*** |
|  | (0.1133) | (0.1786) | (0.5260) | (0.1824) | (0.2217) |
|  | 1.09, 1.54 | 0.85, 1.55 | 0.15, 2.21 | 0.80, 1.51 | 1.42, 2.29 |
| Constant | -2.7232*** | -2.5430** | -2.0544 | -3.1480*** | 0.4670 |
|  | (0.6549) | (1.0893) | (3.9105) | (1.2181) | (1.3156) |
|  | -4.01, -1.44 | -4.68, -0.41 | -9.72, 5.61 | -5.54, -0.76 | -2.11, 3.05 |
| *N* | 6444 | 1770 | 547 | 1944 | 2045 |
| pseudo *R*^2^ | 0.284 | 0.198 | 0.456 | 0.232 | 0.317 |
| p | 0.0000 | 0.0000 | 0.0000 | 0.0000 | 0.0000 |

Source: Authors’ Calculations. Note: *** is significant at p<0.01, ** is significant at p<0.05, * is significant at p<0.10.

Figures in parentheses are standard errors with those not in parentheses but following the standard errors being confidence

Intervals. NSCPHGW, NSCPHFT are non-self cluster proportion of households with good water, non-self cluster proportion of households with flush toilet respectively. To account for women without Partners, a category was created for missing

husbands under partner’s education but coefficient not reported.
